# Integration of Fibroblast-Populated Collagen Lattices and Perfusable Micro-Physiological Systems: A Mechanobiologically Unified Framework for Living Devices

**DOI:** 10.3390/mi17020171

**Published:** 2026-01-28

**Authors:** Kawmini Appuhami, Aya Nakamura-Norimoto, Yasuyuki S. Kida

**Affiliations:** 1 School of Comprehensive Human Sciences, Life Science Innovation, University of Tsukuba, Tsukuba 305-8572, Japan; 2 Cellular and Molecular Biotechnology Research Institute, National Institute of Advanced Industrial Science and Technology (AIST), Tsukuba 305-8565, Japan; 3 School of Integrative and Global Majors, University of Tsukuba, Tsukuba 305-8572, Japan

**Keywords:** micro-physiological, mechanobiology, integration

## Abstract

This review proposes mechanical crosstalk between stromal tension and vascular shear/flow as a unifying principle for integrating fibroblast-populated collagen lattices (FPCLs) with perfusable micro-physiological systems (MPSs). We argue that current in vitro platforms either emphasize fibroblast-driven matrix contraction (as with FPCLs) or flow-mediated vascular dynamics (as with MPSs) but rarely consider the reciprocity between these forces. By defining a mechanobiological framework that couples cellular contractility, extracellular matrix (ECM) remodeling, and shear-dependent endothelial responses, we reframe FPCL–MPS hybrids as “living devices” capable of capturing mechano-transduction across stromal and vascular compartments. This review (1) delineates the mechanobiology of FPCLs, highlighting their tension generation, matrix remodeling, and disease relevance; (2) surveys perfusable MPS design principles, focusing on shear stress, barrier function, and multicellular integration; (3) formulates a crosstalk paradigm in which stromal tension and vascular shear coregulate tissue physiology; (4) synthesizes engineering strategies for integrating FPCLs into MPSs; and (5) outlines challenges and future directions involving multiscale measurements, multi-omics, artificial intelligence, and regulatory standardization. To our knowledge, this review is among the first to explicitly frame stromal tension and vascular shear as a unified mechanobiological axis.

## 1. Introduction

### 1.1. Defining the Scope of the Review and Conceptual Gaps in the Literature

The scope of this review is shaped by two conceptual gaps in the current literature. Although FPCL mechanobiology and MPS engineering have each been reviewed in detail, no studies have clearly explained how mechanical forces in the stromal and vascular domains interact. The first gap concerns the separation of mechanical stimuli. FPCL studies demonstrate how fibroblast contractility generates tension and reorganizes collagen, while MPS research focuses on shear-dependent endothelial biology. Only a few models consider their reciprocal feedback—for example, how stromal compaction modifies microchannel geometry and shear stress, or how flow-induced shear influences fibroblast phenotype and ECM remodeling. The second gap concerns the limited strategies for integrating FPCL constructs into perfusable platforms. Achieving this requires reconciling the behavior of soft contractile matrices with the constraints of microfabricated channels, yet existing hybrid systems remain largely descriptive and do not offer a unifying mechanobiological framework. This review therefore addresses mechanical crosstalk between stromal tension and vascular shear as its central thesis by conceptualizing FPCL–MPS integration as the coupling of two interdependent force fields, tissue tension and fluid shear, which together provide a foundation for interpreting and designing future living devices. While reviews on FPCL mechanobiology have detailed fibroblast-driven matrix remodeling [1], and organ-on-a-chip reviews have focused on perfusion and endothelial biology [2], none have integrated these perspectives into a unified mechanobiological framework.

### 1.2. Organ-on-a-Chip/Micro-Physiological Systems (MPSs)

Organ-on-a-chip and micro-physiological systems (MPSs) represent a developing category of in vitro models that reiterate key physiological and mechanical features of human tissues within miniaturized and perfusable platforms. These systems integrate biological components such as extracellular matrices (ECMs) and living cells with microfabricated devices capable of yielding precise chemical, mechanical, and fluidic stimuli [3,4,5]. MPSs enable more accurate modeling of organ function, disease progression, and responses to pharmacological interventions, with modern MPSs also embedding biosensors and real-time monitoring systems, allowing high-definition characterization of cellular responses under conditions closely resembling physiological environments [2,3]. Beyond single-organ studies, multi-organ MPS networks are employed to investigate interorgan interactions, drug metabolism, and systemic toxicity, bridging the critical gap between in vitro experiments and human biology [6,7]. The 2D and 3D monoculture of cells followed by animal testing represents the traditional experimental model for preclinical phases of drug development. However, such systems do not generally reproduce the complexity and dynamism of human physiology, and there is a mismatch between preclinical and clinical results. To overcome this translational divide, organ-on-a-chip or micro-physiological systems have been developed into sophisticated in vitro models, synthesizing cell biology, micro-engineering, and tissue engineering concepts to recreate functional and human-relevant microenvironments. MPSs also have microfluidic channels that mimic blood and nutrient exchange, unlike cultures, which neither provide real-time physiological responses or continuous perfusion nor regulate shear stress and physiological responses [8,9].

Recent advances in the development of MPSs have led to models that recapitulate the physiology and pathophysiology of organs such as the liver, lungs, gut, brain, and vascular barriers. A recent study developed a liver-on-a-chip system incorporating human iPSC-derived hepatocytes and endothelial cells encapsulated within a 3D hydrogel matrix. Under dynamic perfusion, this platform enhanced key liver-specific functions and supported more physiologically relevant drug metabolism. These findings indicate that such micro-physiological systems can contribute to hepatotoxicity and pharmacokinetic assessments, although they cannot yet completely replace established in vitro or in vivo pharmacokinetic models [9]. Another study reveals that biomechanical stresses such as shear stress, matrix integrity, and cyclic stretch play important roles in cell regulation in vivo. The integration of physical stimuli into organ-on-a-chip systems facilitates closer recapitulation of tissue function and disease pathways. Moreover, the incorporation of patient-derived or genetically engineered cells would enable MPSs to reproduce inter-individual variability, supporting more personalized in vitro testing approaches, although these systems are not yet clinically validated for precision medicine [10,11].

The integration of mechanobiology into micro-physiological systems is becoming critical to translational research and regulatory compliance. The growing adoption of organ-on-a-chip platforms in early-stage drug development reflects their potential to improve predictive accuracy and reduce reliance on animal models [12]. Moreover, regulatory initiatives such as New Approach Methodologies (NAMs) emphasize the need for physiologically relevant, reproducible in vitro systems that can support safety assessments and provide mechanistic insights. Technological advances in bioprinting and sensor integration now enable real-time monitoring of mechanical cues, making it feasible to incorporate stromal tension and vascular shear into perfusable chips [13]. These developments underscore the urgency of establishing unified frameworks that link cellular contractility, extracellular matrix remodeling, and flow-mediated signaling, transforming descriptive models into predictive platforms for precision medicine.

### 1.3. FPCL Model and Its Biological Significance

In parallel to an MPS, a fibroblast-populated collagen lattice (FPCL) represents a mechanobiologically active 3D tissue platform. It is a classical in vitro framework that significantly impacts the active interactions between fibroblasts and the extracellular matrix, specifically collagen. Initial investigations found that contracted FPC is largely caused by three interdependent cellular processes: direct cellular contraction, tractional forces produced during cell locomotion, and preliminary elongation due to the spreading of fibroblasts in the collagen network. As a result, fibroblasts apply mechanical forces to collagen fibrils and rearrange and compact the lattice to generate quantifiable contraction. The model can therefore be used as a predictable platform to investigate cell-mediated mechanical forces and their role in tensioning and remodeling tissue [1].

Over time, FPCL models have been modified to replicate complex microenvironments like the tumor stroma. These systems include fibroblasts that are sown in collagen gel, which can respond to mechanical and biochemical cues to differentiate into cancer-associated fibroblasts. The 3D collagen architecture supports physiologic cell–cell and cell–matrix interactions and can reproduce key microenvironmental features, including matrix stiffness and, in some cases, hypoxic regions that arise from diffusion limitations within the dense gel [14]. Collagen lattices were originally explored as components of early skin-equivalent constructs for burn grafting, but they proved clinically ineffective as permanent grafts. Fibroblast-populated collagen lattices (FPCLs), developed from these early lattice systems, subsequently became important research models rather than therapeutic products. In FPCLs, fibroblasts generate actin-dependent traction forces that reorganize and compact collagen fibrils, resulting in lattice contraction through matrix remodeling and associated fluid displacement [15].

FPCLs have improved our understanding of fibroblast-driven matrix remodeling and the theoretical basis of wound contraction, demonstrating how coordinated cell–matrix and cell–cell interactions regulate collective contractile behavior. Standard FPCLs typically show concentric contraction with randomly oriented fibroblasts, limiting their resemblance to organized scar tissue. Recent studies have explored improving FPCL models through controlled mechanical cues and directional guidance, while microfluidic integration remains an emerging experimental approach rather than an established practice in the field [16,17].

### 1.4. Why Existing Models Fail Mechanically

Traditional cell cultures employed 2-dimensional (2D) monoculture to study cell biology, disease mechanisms, and drug responses. In such systems, cells are cultivated on flat plastic or glass surfaces, which offer simplicity and reproducibility [18]. However, 2D cultures do not simulate the mechanical and structural complexity of living tissues. Two-dimensional cell cultures exhibit altered morphology and polarity, non-physiological gene expression, and limited realistic interactions with the intra- and extracellular matrix [19]. Three-dimensional (3D) cultures were developed to overcome these limitations of 2D systems by providing a more physiologically relevant microenvironment. In these models, cells are cultured on scaffolds or hydrogels and can self-organize into multicellular aggregates that recapitulate aspects of tissue architecture. This configuration allows more natural cell–cell and cell–matrix communication and predicts drug responses more accurately than 2D cultures. However, most current 3D models are not dynamic and do not possess the flow nutrient gradients and mechanical forces of living tissues [20]; because these systems lack intrinsic flow or movement, they do not inherently reproduce physiological processes that depend on perfusion or mechanical cues (for example, shear stress or cyclic strain), although such stimuli can be applied using external devices,.

There are certain complications associated with static 3D cultures, such as uneven nutrient and oxygen distribution, which may lead to cell death in high-density regions. Thus, while 2D and static 3D systems continue to be useful tools, they cannot fully replicate the dynamics and functions of the human tissue environment; therefore, more advanced perfused culture systems are needed [18,19]. Recent reviews on organ-on-a-chip technologies emphasize their ability to replicate physiological flow and biochemical gradients, but they rarely address stromal mechanics as a co-regulatory factor [4].

This table outlines the key differences between traditional 2D monolayer cultures and static 3D culture systems in terms of geometry, cell behavior, mechanical cues, and translational relevance (Table 1). While 3D models improve tissue-like architecture and cell–cell/cell–matrix interactions, they remain diffusion-limited and lack intrinsic perfusion-driven mechanical stimuli, necessitating the development of perfusable micro-physiological systems [18,19,20].

Although micro-physiological systems (MPSs) mimic micro-physical features, many platforms still lack certain mechanical signals needed to support cellular behavior and matrix remodeling. FPCLs offer a micromechanical niche in which cells contract, remodel, and organize extracellular matrix fibers [7,21,22]. By combining micromechanical with microfluidic platforms, controllable flux forces and mechanical forces are used to replicate in vivo forces. This combined method includes mechanical and biochemical stimuli, which is essential to achieving realistic tissue characterization. Thereby, recreating the combination of tissue perfusion and tension enhances the system’s physiological relevance, allowing more realistic tissue behavior modeling, wound healing, fibrosis, and drug responses.

FPCLs like micromechanical model constructs could be placed in microfluidic devices to provide continuous perfusion and accurate biochemical gradients [23]. However, FPCL behavior depends on cell density and substrate adhesion.

Additionally, micromechanical systems with perfusing flow enable drug testing under physiological conditions and provide insight into how mechanical forces influence tissue responses, drug efficacy, and toxicity, though their throughput remains lower than that of conventional 2D or spheroid screening platforms. This kind of fusion improves reproducibility, decreases the use of animal models, and bridges the gap between the static 3D cultures and fully functional tissue-on-a-chip models.

Despite widespread acknowledgment that cells integrate multiple mechanical inputs, few studies derive explicit force transfer pathways linking fibroblast traction to lumen geometry and shear amplification, or couple these pathways to endothelial signal transduction under flow. This analytical gap is of clinical importance, because fibrotic progression, desmoplastic tumor biology, and impaired wound resolution each emerge from crosstalk between stromal contractility, extracellular matrix stiffening, and vascular dynamics. FPCL–MPS hybrids enable controlled analysis of mechanical crosstalk by combining a contractile collagen lattice with perfusable microchannels, allowing assessment of lattice compaction, the resulting lumen changes, shear redistribution, and key signaling responses in fibroblasts and endothelia [14,24]. Recent tumor stroma FPCL models and fibrosis-on-chip systems illustrate application-ready contexts where coupling these mechanics can clarify drug responses and resistance mechanisms under dynamic flow [14]. This review advances this field of research by formalizing a unified mechanobiological perspective, arguing for quantified sensor-integrated designs that move beyond descriptive hybrids toward predictive living devices [25].

## 2. Stromal Mechanics in Fibroblast-Populated Collagen Lattices

### 2.1. Principles of FPCL Mechanics

Fibroblast-populated collagen lattices recreate dynamic cell–ECM coupling in a fibrillar collagen network, where fibroblasts generate and transmit contractile forces to the matrix through integrin-mediated adhesions linked to actomyosin machinery. Traction progressively compacts and reorganizes the lattice, providing an in vitro analog of wound contraction and early granulation tissue formation [26]. As tension accumulates, fibroblasts sense rigidity and deformation and adapt their spreading and contractility, establishing bidirectional feedback between cellular force generation and matrix mechanics that is not reproduced by passive hydrogels [26,27]. Contraction kinetics are density-dependent. At a moderate cell density, the lattice compacts more slowly and exhibits organized collagen remodeling, whereas at a high density, compaction is rapid and fibril order is reduced. Together, these features establish FPCLs as living force transducers that connect single-cell traction to emergent tissue-scale mechanics in a way that scaffold-only systems cannot [1,21].

### 2.2. Unique Contributions of FPCL to Force Generation

Fibroblast-populated collagen lattices (FPCLs) provide mechanical capabilities that passive hydrogels cannot. They generate endogenous, sustained tension without external actuators, as fibroblasts continuously convert actomyosin forces into lattice compaction, modeling wound contraction, and stromal stiffening as emergent phenomena rather than imposed loads [1,15,26]. This tension drives closed-loop load–remodeling feedback, where collagen mechanics regulate fibroblast spreading, contractility, and differentiation, reinforcing matrix reorganization in a way that static gels cannot [27,28]. FPCLs also enable density- and adhesion-dependent tuning of hydraulic access and shear transmission, since compaction rate and fiber alignment dictate permeability and shear stress propagation from perfused channels [22,29]. When integrated with microfluidic systems, FPCLs form a mechanically truthful interface, where endogenous tension reshapes lumen geometry, redistributes shear, and triggers endothelial mechano-signaling revealing causal pathways that passive matrices cannot replicate [30,31]. These properties underpin application leverage under realistic load, allowing FPCL–MPS hybrids to assess antifibrotic and anti-invasive drug responses and model tumor–stroma reciprocity under perfusion with coupled mechanical regulation [14,24,32]. Conceptually, FPCL contraction can be viewed as a force balance between cell-generated actomyosin tension and collagen network resistance [15,27]. As compaction proceeds, permeability decreases, altering local interstitial flow [33], and also altering shear transmission when coupled to perfused channels [2,34].

Previous experimental and modeling studies have estimated FPCL-generated mechanical stresses to be in the order of 10^2^–10^3^ Pa [28], depending on fibroblast density, collagen concentration, and boundary constraints. Importantly, these stress levels develop over hours to days, overlapping the timescales of ECM remodeling and perfusion-driven endothelial adaptation in micro-physiological systems [14].

### 2.3. Matrix Remodeling and 3D Architecture

An FPCL is a model of mechanobiology, providing a 3D framework that can respond to how fibroblasts remodel and organize the extracellular matrix by means of self-generated contractile forces. This self-contraction is caused by tension on the actin–myosin crossbridge, mediated by integrin-mediated adhesions that cause collagen fibrils to align and the fluid to expel, resulting in a mechanically active 3D micro-architecture that resembles aspects of early wound healing and granulation tissue formation [1,26]. This concept has been further refined, with some studies demonstrating that remodeling of the matrix in an FPCL is a dynamic feedback process whereby fibroblast activity and ECM mechanics regulate each other. As strain builds up in the lattice, fibroblasts modulate morphology and contractility, and alternate between quiescent and biosynthetically active states, based on matrix rigidity and mechanical forces [27].

**Figure 1:**
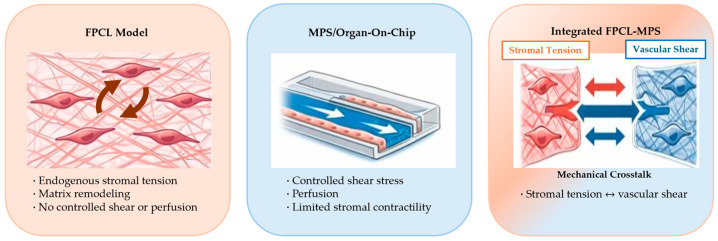
Conceptual gap in current in vitro tissue models and the motivation for FPCL–MPS integration. Fibroblast-populated collagen lattice (FPCL) models emphasize endogenous stromal tension and matrix remodeling driven by fibroblast contractility but lack controlled vascular perfusion and shear stress. Microphysiological systems (MPSs) and organ-on-chip platforms enable precise regulation of flow and endothelial shear stress but often treat the surrounding extracellular matrix as mechanically passive. Integration of FPCLs with perfusable MPS establishes a hybrid platform in which stromal tension and vascular shear interact bidirectionally, providing a mechanobiologically unified framework for living devices. Generated with Chat GPT 5.2.

**Figure 2:**
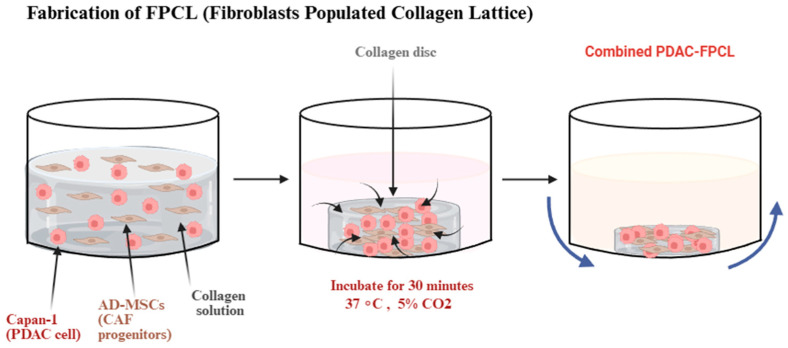
Graphical representation of the establishment of comPDAC-FPCL models (with Capan-1 and CAF progenitors embedded in Collagen solution). The blue arrows show the rotation direction of cells incubated in a shaker. Created in BioRender. Kawmini, A. (2026). https://app.biorender.com/illustrations/691d6d1ed4d1b390ea9ea68d.

The 3D FPCL model of Pancreatic Ductal Adenocarcinoma has been able to recapitulate the physical and biological aspects of the tumor microenvironment. It represents a 3D co-culture system designed to replicate the fibrotic and mechanically stressed PDAC microenvironment. This model is constructed by embedding PDAC epithelial cells called Capan-1, as well as cancer-associated fibroblast (CAF) progenitors derived from adipose-derived mesenchymal stem cells, within a type-I collagen matrix, forming an FPCL (Figure 1). Regarding the latter, it has been discovered that heterogenous CAFs in the collagen lattice are further subdivided into discrete subtypes and assume spindle-shaped morphologies around glandular pancreatic cancer structures with a clinical PDAC architecture. Notably, drug screening (Figure 2) revealed that therapies targeting both cancer cells and stromal CAFs were more effective than treatments targeting cancer cells alone, indicating that the stroma contributes directly to chemoresistance. These results demonstrate that the FPCL model is physiologically relevant for investigating CAF–cancer interactions, tumor mechanics, and therapeutic responses [14].

### 2.4. Physiological Relevance and Limitations

The FPCL model offers distinct advantages in replicating physiological tension and the mechanical microenvironment observed in vivo. Unlike static 2D cultures, FPCLs enable fibroblasts to generate and transmit contractile forces through a three-dimensional collagen network, closely simulating the dynamic mechanical cues of native tissues. This self-contraction process establishes tensional homeostasis, allowing cells to sense and respond to matrix stiffness, density, and deformation.

Studies have shown that fibroblasts embedded in collagen lattices exert contractile forces that reorganize the matrix, providing intrinsic tissue tension like that in wound healing environments. In addition, fibroblast traction and spreading, as well as myofibroblast differentiation, generate sustained tension and matrix compaction, producing a mechanically dynamic 3D structure [1,26]. In cancer models such as the comPDAC-FPCL, this mechanical feedback promotes the formation of fibrotic stroma and CAF heterogeneity, paralleling the desmoplastic reactions found in pancreatic tumors [14].

Collectively, these findings highlight that FPCLs capture selected mechanical feedback loops, allowing us to study how tension, stiffness, and ECM remodeling shape cellular phenotypes, tumor progression and therapeutic responses.

## 3. Vascular Shear Mechanics in Perfusable Micro-Physiological Systems

### 3.1. Design and Principles of Perfusable MPS Devices

Perfusable micro-physiological systems have developed as next-generation in vitro platforms that integrate microfluidic perfusion, engineered extracellular matrices (ECMs) and muticellular organization to enhance the representation of physiological tissue behavior, while still functioning as simplified approximations of in vivo tissues. The principle of this system relies on continuous perfusion through embedded vascular-like channels, which maintain nutrient and oxygen supply while generating the mechanical cues needed for cellular homeostasis, such as shear stress and interstitial flow. Incorporating endothelial lining, ECM stiffness, and biochemical gradients enhances the physiological relevance of MPSs, though these systems do not fully recapitulate the in vivo microenvironment [30,35].

A recent study on perfused 3D tissue developed a tubular, liver-like construct capable of continuous perfusion. This model featured central microchannels surrounded by capillary-like networks that self-organized under flow conditions. The presence of flow triggered angiogenic-gene activation, supported structural maturation, and enhanced functional outputs such as albumin secretion, demonstrating how mechanical cues from perfusion can drive both vascular remodeling and tissue functionality [36].

### 3.2. Multi-Organ Coupling and Sensor Integration

The authors of [37] created a coupled small intestine–liver device that allowed direct perfusion between these two tissues. This in vitro device promoted metabolic communication, boosted hepatocyte CYP enzyme activity, and strengthened the intestinal epithelial barrier, providing a platform for studying multi-organ interactions under controlled laboratory conditions. In addition, recent work found that sacrificial bioprinting can be employed to create vascularized 3D tumor models, enabling perfusion-driven endothelial growth and providing a dynamic platform to study early metastatic behavior [38]. Complementing this, another study developed a tumor-on-a-chip system to reveal how intraluminal flow influences the proliferation of cancer cells and their response to therapy within a continuously perfused environment [39].

Based on these biological advances, another study developed a poly (ethylene glycol)-based engineered extracellular matrix platform to support de novo vasculogenesis and create interconnected, perfusable microvasculature. By integrating pressure and flow stimuli with paracrine signaling across a nanoporous silicon-nitride membrane, the system forms a modular micro-physiological platform that recapitulates key aspects of tissue-specific vascular niches, such as the choriocapillaris, without fully reproducing in vivo complexity [31]. Similarly, recent investigations found that modern MPS technology, which now incorporates clinically derived organoids, stem cell-based models, and circulation sensors, is advancing cancer research, although its widespread clinical translation remains limited. These novel technologies enable real-time monitoring of tumor–stroma interactions, immune cell infiltration, and drug responses [33].

Extending beyond single-organ systems, recent advances demonstrate that multiple-organ modules such as the gut, liver, and cardiac tissues can be interconnected through microfluidic circulation to reproduce physiologically relevant organ–organ communication. These multi-organ platforms support coordinated metabolic activity, barrier function, and systemic responses that isolated units cannot achieve. In addition, incorporating embedded biosensors such as oxygen, pH, glucose, and TEER sensors enables continuous functional readouts and improves real-time monitoring of integrated tissue performance [40,41,42].

Advanced fabrication strategies such as design-encoded 4D printing have recently emerged to address the challenge of creating vascular grafts with complex geometries and cell compatibility. Using shape-memory polymers with anisotropic design encoding, flat sheets can be programmed to self-roll into hollow tubes under mild thermal triggers and subsequently recover their permanent shape at physiological temperatures. This dual shape-morphing capability enables precise control over graft dimensions and facilitates endothelial cell seeding in temporary flat configurations before reconfiguration into tubular forms, offering a promising route for patient-specific, bioresorbable vascular grafts with improved long-term patency compared to conventional electrospun or molded constructs [43].

### 3.3. Limitations and Opportunities

While MPSs have revolutionized perfusion culture, challenges remain. Some devices rely on rigid polymers that do not deform under cell-generated forces, limiting mechano-transduction [44], and media recirculation can introduce uncontrolled gradients of cytokines and metabolic byproducts. Additionally, long-term cultures may experience microchannel occlusion due to ECM deposition or cellular overgrowth. Most critically, MPSs rarely incorporate contractile stromal elements capable of generating tension, leaving a mechanobiological gap between flow and matrix mechanics [25,45].

Collectively these innovations highlight how rapidly perfusable MPS technology is expanding with engineering precision and biological realism, advancing the development of mechanically active, functionally perfused, and physiologically relevant in vitro tissue systems that more effectively model aspects of human biology [36,37,38,39].

## 4. Mechanical Crosstalk: Coupling Stromal Tension and Vascular Shear

**Figure 3:**
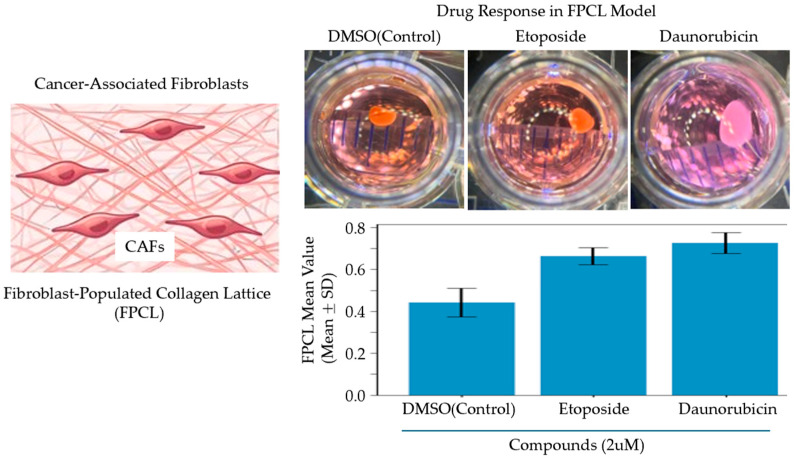
Representative FPCL-based assessment of chemotherapeutic response. A fibroblast-populated collagen lattice (FPCL) containing cancer-associated fibroblasts (CAFs) was used to assess drug response. Representative FPCL images and cell viability measurements following treatment with DMSO (control), etoposide, or daunorubicin (2 µM) are shown. Differences in viability across compounds illustrate the contribution of the FPCL stromal environment to drug response. Values represent mean ± SD.

The central premise of this review is that stromal tension and vascular shear are not independent; they continuously influence one another in living tissues. We propose a conceptual framework in which FPCL and MPS components interact through reciprocal mechanical signals (Figure 3).

**Figure 4:**
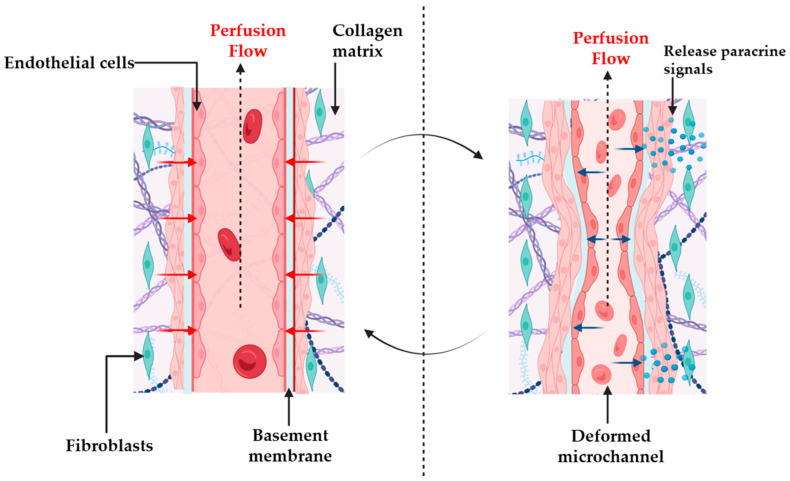
A conceptual diagram illustrating mechanical cross-talk in FPCL–MPS hybrids. A contractile fibroblast-populated collagen lattice generates tension (short red arrows) that compresses and deforms adjacent microchannels (**left**). Perfusion through these channels places shear stress on endothelial cells (short dark blue arrows), which in turn release paracrine signals (**right**) that modulate fibroblast behavior. Matrix stiffness influences endothelial mechano-transduction, while shear stress alters fibroblast differentiation. The arrows depict reciprocal interactions that constitute a feedback loop. Created in BioRender. Kawmini, A. (2026). https://app.biorender.com/illustrations/692d62a88836ddcb525ee86f.

**Figure 5:**
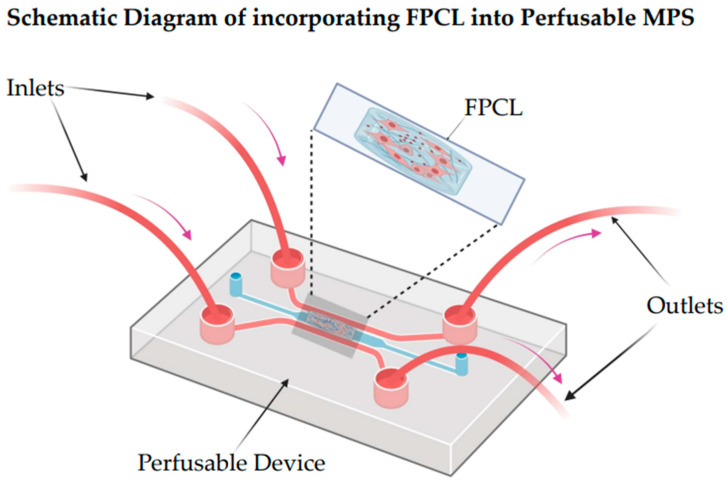
A schematic 2D illustration of how a fibroblast-populated collagen lattice (FPCL) can be incorporated into a perfusable micro-physiological system (MPS). The FPCL construction is positioned within the central chamber of a transparent perfusion device, enabling controlled fluid transport across the tissue model. Pink arrows denote the direction of perfusion flow, originating from the inlets and exiting through the outlets, highlighting the integration of the FPCL with the device’s microfluidic architecture. This conceptual representation emphasizes the potential for combining FPCL-based tissue models with perfusable platforms to enhance physiological relevance in in vitro studies. Created in BioRender. Kawmini, A. (2026). https://app.biorender.com/illustrations/6928414d4053d0434933eb7c.

Introducing FPCLs into MPSs represents an effective pathway to assembling living devices that enable a transition from classical tissue constructs to active organ-on-a-chip technology (Figure 4). Thanks to their self-contractile and mechanically responsive 3D collagen matrix, FPCLs can be placed into microfluidic chambers or perfusable scaffolds, allowing the real-time regulation of mechanical tension, matrix remodeling, and fluid flow. This integration could be carried out by microfabricating collagen lattices in perfusion channels using soft-lithography techniques or micro-molding (Figure 5) [31,33]. The collagen lattice would also constitute a tunable ECM compartment that is seeded with fibroblasts or co-cultured cells, while the surrounding microchannels would enable nutrient delivery through controlled perfusion and the application of fluid shear stress.

By incorporating FPCLs into this type of perfusable system, researchers can simultaneously regulate tensional forces and hydrodynamic signals, replicating the dynamic reciprocity between cellular contraction, ECM remodeling, and tissue-level mechanics (features of wound healing), as well as fibrosis and modeling of the tumor microenvironment. The hybrid FPCL-MPS design enables biomechanical stimuli to be precisely modulated, and allows researchers to study the role of matrix tension and interstitial flow in the joint regulation of fibroblast differentiation and ECM reorganization. Micro-actuated/flexible chip designs can be used to create cyclic strain or pressure differentials to simulate in vivo tissue tension [46], and microfluidic perfusion can be used to produce oxygen, nutrient, and signaling molecule gradients in the collagen matrix. Moreover, tension–flow co-regulation has also been predicted to play a role in fibrotic remodeling, in which a dynamic interaction of sustained myofibroblast contractility (as observed in PFCLs) with endothelial and epithelial perfusion can model aspects of fibrosis progression in vitro [24,47].

The changes in shear stress and flow direction that are enabled by reconfigurable perfusion devices [48] can be applied to FPCL systems to examine aspects of endothelial or stromal responses such as matrix remodeling or fibrotic stiffening when relevant cell types are incorporated. Introducing FPCLs into microchips that allow for easy integration enables their application in not only mechanobiology, but also translational and precision medicine. FPCL-MPS hybrids can model the aspects of fibrotic micro-physical environments; for example, in cardiac fibrosis-on-a-chip systems, fibroblasts and cardiomyocytes are co-cultured to network biomechanical and structural phenotypes of fibrosis and optical or mechanical measurements of contractility and stiffness [24].

Similarly, tumor–fibroblast interactions can be simulated using FPCL-like collagen matrices that are loaded with cancer-associated fibroblasts (CAFs) and tumor spheroids in microfluidic systems, enabling researchers to investigate reciprocal paracrine signaling, tumor invasion, and drug resistance [32,49]. Transplantation of collagen microtissues in hepatic systems has been effective in preserving primary hepatocyte functions over prolonged durations, with significant physiological relevance in drug metabolism and toxicity screening [50].

In addition, studies have shown that FPCL matrices can be further improved by incorporating microchannels lined by endothelial cells or self-assembled vascular networks, which not only promote perfusion, but are also physiologically relevant and support angiogenesis and nutrient exchange [51,52]. This cross-linking of FPCL-based tissues with biohybrid MPSs suggests the creation of living devices—systems that incorporate cellular contractility, dynamic ECM remodeling, and real-time sensing in micro-engineered devices. Increased integration of bioelectronics and soft materials now makes it possible to create living tissue interfaces capable of recording or controlling the behavior of cells via implanted sensors or actuators, though their stable, fully functional integration remains challenging [53].

Moreover, FPCL tissues can be produced in patient-specific forms using 3D bioprinting and organ-building-block (OBB) approaches to create patient-specific tissues (in terms of form and function) and turning them into perfusable and vascularized structures. Multi-organ coupling systems can connect such tissues to model selected aspects of systemic disease or drug handling, although they do not replicate full-body pharmacokinetics [54,55,56]. The combination of these innovations has established the basis of next-generation biomimetic systems that move beyond static in vitro cultures and exhibit more dynamic, context-dependent responses, though fully adaptive, self-regulating, physiologically integrated systems have not yet been achieved.

### Mechanistic Sub-Model with Indicative Scales and Pathways

In fibroblast-populated collagen lattices (FPCLs), endogenous actomyosin traction drives lattice compaction over hours to days, routinely quantified as reductions in gel area or thickness, and accelerated by higher cell density or lower substrate adhesion [1,15,22,29]. The tension generated within FPCLs is generally in a low Pascal range of ∼3–80 Pa (typically ≈15 Pa), reflecting the balance between cellular contractility and the mechanical resistance of type-I collagen gels [57,58]. As a contracted FPCL abuts a perfused microchannel, ECM compaction translates into lumen narrowing, and under steady flow, this geometry change predictably amplifies endothelial wall shear. In perfusable micro-physiological systems (MPSs), endothelial shear bands of ~1–20 dyn·cm^−2^ (dyne per square centimeter, a unit of shear stress and force per area) are commonly applied and considered physiologic, so a 10–30% effective diameter decrease can shift channels from low-shear to a more mechano-active regime [2,59,60]. This shear amplification engages canonical flow-responsive signaling, including KLF2/KLF4–eNOS via ERK5-linked mechano-sensors (P2 × 4; PIEZO1), and YAP/TAZ modulation under disturbed vs. laminar flow [61,62,63,64]. In application-ready contexts, FPCL tumor stroma and vascularized tumor-on-chip platforms demonstrate how stromal tension and perfusion-dependent shear co-regulate invasion, drug penetration, and endothelial phenotypes under dynamic flow [14,39].

## 5. Integrated FPCL–MPS Platforms: Design Principles and Applications

### 5.1. Engineering Strategies

Creating living devices that embody mechanical crosstalk requires careful integration of contractile matrices and perfused microchannels. Existing attempts to merge collagen-based or FPCL-like lattices with perfusable micro-physiological systems can be grouped into several design patterns.

One approach positions collagen or FPCL-like matrices within static or porous scaffolds adjacent to perfusion channels. These systems enhance nutrient transport and allow partial coupling between lattice remodeling and perfused microenvironments. Studies have also revealed that porous collagen gels can sustain tumor–stroma communication under flow, although the lack of endothelial interfaces and limited mechanical reciprocity restrict their physiological relevance [65,66].

Progress in microfabrication and bio-MEMS (Micro-Electro-Mechanical Systems) technologies, such as soft lithography, micro-molding, 3D bioprinting, and sacrificial templating, has made it possible to build miniaturized reproducible and sensor-integrated micro-engineered platforms, an area of growing relevance to micro-machine researchers [30,54].

Another existing design pattern embeds collagen matrices directly inside microfluidic channels that are subsequently endothelialized. This design supports controlled shear stress, angiogenic sprouting, and endothelial–stromal signaling. It shows that endothelialized collagen channels respond dynamically to flow, but the collagen often behaves as a passive hydrogel rather than a contractile FPCL due to geometric constraints and shear-induced stabilization [34].

Another approach uses bioprinting or sacrificial templating to create perfusable, vascularized constructs embedded within collagen or hybrid ECMs. These systems enable reproducible vascular networks and long-term perfusion. Studies have also demonstrated that sacrificially printed microchannels can be integrated into dense ECM hydrogels, though printability requirements often limit fibroblast-mediated tension and authentic FPCL-like remodeling [67,68].

### 5.2. Advantages of Hybrid FPCL-MPS Devices over Standalone Platforms

**Figure 6:**
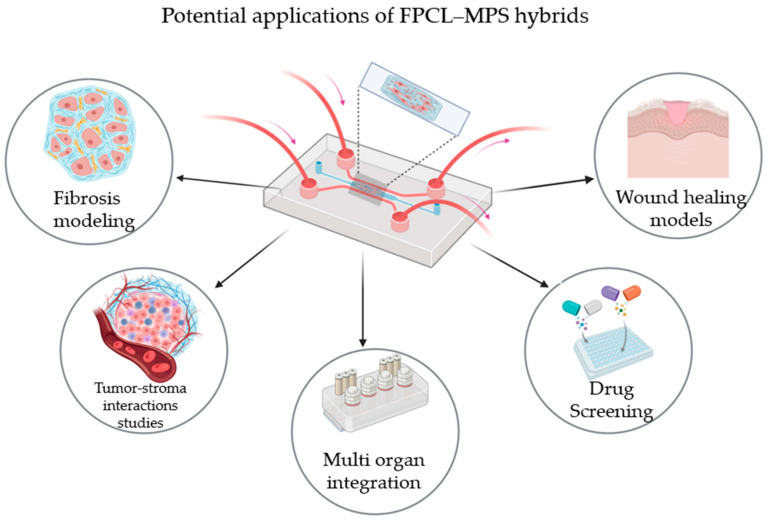
A schematic illustration of the central FPCL–MPS hybrid platform and its diverse applications. The hybrid system combines fibroblast-populated collagen lattices with a perfusable microfluidic architecture to enable the establishment of physiologically relevant models. The surrounding illustrations represent key application areas: fibrosis modeling, wound healing studies, tumor–stroma interaction analysis, drug screening platforms, and multi-organ integration. These applications leverage mechanical crosstalk between stromal tension and vascular shear to improve disease modeling and therapeutic testing. Created in BioRender. Kawmini, A. (2026).

Hybrid FPCL–MPS devices offer capabilities that neither platform provides on its own (Figure 6). One major advantage is their ability to capture reciprocal mechano-transduction. These systems make it possible to study how stromal contraction alters perfusion patterns and how fluid shear influences fibroblast behavior, enabling the study of reciprocal signaling pathways that cannot be observed in isolated models.

Another key feature is the establishment of physiological gradients. Continuous perfusion establishes oxygen, nutrients, and cytokine gradients within the matrix, closely mimicking in vivo microenvironments [4,30,35,54]. Hybrid platforms support increased multicellular complexity. By co-culturing fibroblasts, endothelial cells, immune cells, and parenchymal cells within the same device, researchers can examine both cell–cell and cell–matrix interactions under mechanically dynamic conditions, as demonstrated in multiple studies involving vascularized or stromal-integrated chip stimulation in a controlled and integrated setting [32,39,49]. These hybrid systems enable advanced drug testing and disease modeling. By tuning matrix stiffness, tissue tension, shear forces, and biochemical conditions, FPCL–MPS hybrids can model wound healing, fibrotic disorders, tumor invasion, and metastasis, providing versatile platforms for screening antifibrotic or anti-angiogenic therapies [9,34,35,66].

### 5.3. Comparative Evaluation of Current Systems

This table outlines the major design strategies for integrating fibroblast-populated collagen lattices (FPCLs) with perfusable micro-physiological systems (MPS) (Table 2). Each approach is characterized by its method of perfusion control, the degree of stromal mechanical activity it supports, and its suitability for specific applications. The strategies range from simple configurations such as FPCLs positioned adjacent to perfusion channels [56] to more advanced systems embedding FPCLs inside endothelialized microchannels for direct shear–tension interaction [66]. Bioprinted or sacrificially templated vascularized constructs enable long-term perfusion and multicellular complexity [34,67], while hybrid modular platforms with integrated sensors offer dynamic control and real-time mechanobiological feedback [40,41,42]. Together, these approaches illustrate a continuum from passive matrices with limited flow coupling to highly customizable, sensor-enabled systems capable of supporting active tension, controlled shear, and complex disease modeling.

## 6. Challenges and Future Perspectives

### 6.1. Multiscale Mechanobiology and Measurement Technologies

Quantifying mechanical cross-talk at varying scales constitutes a major challenge. Live imaging of matrix deformation, cell traction, and shear stress in real time requires integrated sensors. Innovations that have been made to overcome this challenge include microscale strain gauges, embedded force-sensitive fluorophores, traction force microscopy, and micro-electromechanical systems (MEMS) that measure fluid pressure and flow. Combining these with high-resolution imaging (e.g., multiphoton microscopy) will clarify how local forces propagate through tissues.

### 6.2. Multi-Omics and Computational Integration

Biological mechanics intersect with gene expression, metabolism, and epigenetics, and single-cell RNA sequencing, proteomics, and metabolomics of FPCL–MPS cultures under defined mechanical conditions can reveal pathways regulated by tension and shear. Integrating these data into digital twins—computational models that simulate tissue behavior based on mechanobiological inputs—could predict patient-specific responses to drugs or injuries. In addition, machine learning can identify patterns linking mechanical cues to phenotypic outcomes.

### 6.3. Artificial Intelligence and Real-Time Control

Closed-loop FPCL MPS platforms can integrate real-time sensor feedback with adaptive control algorithms to dynamically regulate mechanical and biochemical cues. Embedded sensors, including oxygen probes, TEER electrodes, pressure sensors, and optical devices for measuring of FPCL contraction, can continuously quantify tissue state and provide inputs for AI-driven controllers that adjust perfusion flow rate, shear stress, cyclic strain, or soluble-factor delivery to maintain predefined physiological targets such as endothelial barrier integrity, stromal tension balance, or controlled matrix stiffening. Reinforcement learning approaches may further optimize system performance by iteratively tuning mechanical inputs to achieve defined phenotypic outcomes, including myofibroblast activation, angiogenic remodeling, or fibrosis-associated extracellular matrix compaction, allowing AI-enabled FPCL MPS platforms to transition from static culture systems toward adaptive living devices capable of reproducing disease progression or maintaining tissue homeostasis.

### 6.4. Standardization and Regulatory Considerations

For FPCL–MPS hybrids to impact precision medicine and drug development, it is essential to standardize fabrication, operation, and data reporting. Variations in collagen source, cell density, flow rate, and mechanical loading hamper reproducibility, so establishing guidelines, proficiency testing, and reference materials will facilitate cross-laboratory comparability. Regulatory frameworks such as New Approach Methodologies (NAMs) and digital-knowledge-based Good Laboratory Practices (DK-GLP) must adapt to accommodate dynamic, mechanically responsive models. Validation against clinical outcomes will be critical for the adoption of FPCL–MPS hybrids.

### 6.5. Towards Multi-Organ Living Devices

The ultimate goal of this field of research is to establish a multi-organ network of FPCL–MPS modules representing the heart, liver, kidneys, lungs, and other tissues, connected by common perfusion channels. Such systems could model systemic pathophysiology, inter-organ communication, and drug pharmacokinetics. However, achieving this will require integrating diverse ECM compositions, mechanical properties, and cell types while maintaining controlled crosstalk. Although this is challenging, incremental progress involving the coupling of two or three modules will improve scalability.

## 7. Conclusions

Fibroblast-populated collagen lattices and perfusable micro-physiological systems have individually advanced our understanding of tissue mechanics and physiology, yet neither captures the full mechanobiological reciprocity inherent to living organs. By positing mechanical cross-talk between stromal tension and vascular shear as the unifying framework for FPCL–MPS integration, this review highlights the transformative potential of hybrid “living devices.” Such platforms enable controlled study of how fibroblast contraction remodels ECM and affects perfusion, and how shear stress and flow regulate stromal behavior. Engineering strategies ranging from microfabrication and bioprinting to sensor integration are converging to enable the successful establishment of these systems. Future work must focus on multiscale measurements, multi-omics integration, AI-driven control, and standardization to accelerate translation into drug testing, disease modeling, and regenerative medicine. Through mechanobiologically unified living devices, we can investigate complex human physiology in vitro and open new avenues for mechanistic discovery and therapeutic innovation.

Practical solutions to address these challenges are emerging. Real-time mapping of tension and shear can be achieved by integrating MEMS-based sensors with advanced imaging techniques; multi-omics integration can combine single-cell sequencing with biomechanical data using cloud platforms for correlation; AI-driven control can apply reinforcement learning to dynamically adjust perfusion and mechanical loading, supported by predictive digital twin models (standardization requires clear guidelines for collagen source, cell density, and flow parameters, along with open datasets for reproducibility); and finally, modular microfluidic connectors and standardized ECM blocks can enable the creation of scalable multi-organ systems validated through pharmacokinetic simulations. Implementing these strategies will help transform FPCL–MPS hybrids into robust platforms for precision medicine and advanced drug discovery.

## Figures and Tables

**Table 1:** Comparison of key features of 2D monolayer culture and static 3D culture systems.

Feature	2D Culture (Monolayer)	3D Culture (Static Hydrogels/Scaffolds)
Substrate and geometry	Flat, rigid plastic/glass; simplified topology	Fibrillar/porous matrices; tissue-like architecture
Cell morphology and polarity	Frequently non-physiological morphology/polarity; altered gene expression	More native-like morphology and polarity; improved lineage markers
Cell-matrix mechanics	Minimal integrin traction transfer; weak mechanoreciprocity	Greater traction transfer and some ECM remodeling, but gels often behave passively (limited mechanical feedback)
Intercellular organization	Planar contact; limited microtissue formation	Multicellular 3D aggregates; richer cell-cell signaling
Transport and gradients	Well-mixed media; no interstitial flow	Diffusion-dominated; uneven O_2_/nutrient distribution in dense regions; hypoxia/necrosis can occur
Dynamic mechanical cues	Shear/strain absent unless externally applied	Intrinsic perfusion is generally absent; shear/strain must be externally imposed; many platforms not deformable

**Table 2:** Summary of the main design strategies used to combine stromal matrices with perfusable platforms.

Design Strategy	Description	Perfusion Control	Stromal Mechanical Activity	Advantages	Limitations	Applications
Adjacent FPCL and Perfusion Channels	FPCL positioned next to microfluidic channels within a chamber	Moderate, relies on diffusion and flow coupling	High (FPCL retains contractility)	Simple assembly; good for tension studies	Limited endothelial interaction; weak shear coupling	Wound healing; models; fibrosis studies
FPCL Embedded Inside Microchannels	Collagen lattice integrated within permeable channels, often endothelialized	High; direct perfusion through channels	Moderate (geometry restricts full contraction)	Enables shear-tension interaction; supports angiogenesis	Risk of channel occlusion; reduced remodeling	Tumor-stroma interaction; vascular remodeling
Bio-printed or Sacrificially Emplated Vascularized Constructs	Perfusable networks printed within collagen or hybrid ECM	High; controlled perfusion and gradient formation	Variable (depends on lattice density and design)	Long-term perfusion; supports multicellular complexity	Complex fabrication; lower throughput	Drug screening; multi-organ chips
Hybrid Modular Systems with Sensors	FPCL integrated with perfusion and embedded sensors for real-time monitoring	High; dynamic control of flow and pressure	High-tension and shear monitored simultaneously	Enables mechanobiological feedback; AI-driven control	High cost; technical complexity	Precision medicine; adaptive tissue models

## Data Availability

The original contributions presented in this study are included in the article. Further inquiries can be directed to the corresponding author.

## Abbreviations

The following abbreviations are used in this review paper:

FPCL	Fibroblast-Populated Collagen Lattice
MPS	Micro-Physiological System
ECM	Extracellular Matrix
CAF	Cancer-Associated Fibroblast
AD-MSC	Adipose-Derived Mesenchymal Stem Cell
TEER	Trans-Endothelial Electrical Resistance
MEMS	Micro-Electromechanical Systems
NAM	New Approach Methodology
